# C-MPL signaling in breast cancer: molecular mechanisms and therapeutic implications

**DOI:** 10.1097/MS9.0000000000004894

**Published:** 2026-04-01

**Authors:** Emmanuel I. Obeagu

**Affiliations:** a Division of Haematology, Department of Biomedical and Laboratory Science, Africa University, Zimbabwe; b Department of Molecular Medicine and Haematology, School of Pathology, Faculty of Health Sciences, University of the Witwatersrand, Johannesburg, South Africa

**Keywords:** breast cancer, c-MPL, JAK/STAT pathway, therapeutic target, tumor microenvironment

## Abstract

The thrombopoietin receptor cellular myeloproliferative leukemia (c-MPL), traditionally recognized for its role in hematopoiesis, has recently emerged as a key mediator of breast cancer progression. Aberrant c-MPL signaling activates multiple oncogenic pathways, including Janus kinase/signal transducer and activator of transcription, phosphatidylinositol 3-kinase/protein kinase B, and mitogen-activated protein kinase/extracellular signal-regulated kinase (ERK), promoting tumor cell proliferation, survival, metastasis, and maintenance of cancer stem-like populations. Elevated c-MPL expression is associated with aggressive tumor subtypes, poor prognosis, and therapy resistance. In addition to tumor-intrinsic effects, c-MPL modulates the tumor microenvironment by enhancing angiogenesis and fostering immunosuppressive conditions. Therapeutic strategies targeting c-MPL, including monoclonal antibodies, small-molecule inhibitors, and combination approaches, show promise in preclinical studies. Understanding the molecular mechanisms underlying c-MPL signaling and its interactions with the tumor microenvironment may facilitate the development of novel targeted therapies and improve clinical outcomes in breast cancer.

## Introduction

Breast cancer is the most frequently diagnosed malignancy among women worldwide and remains a leading cause of cancer-related mortality. Its clinical management is challenged by marked molecular heterogeneity, which influences tumor behavior, prognosis, and therapeutic response^[^1,2^]^. Molecular subtypes – such as hormone receptor-positive (estrogen receptor and progesterone receptor), human epidermal growth factor receptor 2 (HER2)-positive, and triple-negative breast cancer (TNBC) – exhibit distinct biological characteristics and clinical outcomes. Despite advances in early detection, targeted therapies, and adjuvant treatment, recurrence and metastasis continue to significantly impact survival, highlighting the need for novel prognostic biomarkers and therapeutic targets^[^3–5^]^. The thrombopoietin (TPO) receptor, c-MPL, is a type I transmembrane receptor historically recognized for its critical role in hematopoiesis, particularly in regulating megakaryocyte proliferation, differentiation, and platelet production. Cellular myeloproliferative leukemia (c-MPL) binds its ligand, TPO, triggering receptor dimerization and activation of downstream intracellular signaling cascades, including Janus kinase (JAK)/signal transducer and activator of transcription (STAT), phosphatidylinositol 3-kinase (PI3K)/protein kinase B (AKT), and mitogen-activated protein kinase (MAPK)/extracellular signal-regulated kinase (ERK) pathways. These pathways regulate proliferation, survival, apoptosis, and cellular metabolism. While the physiological role of c-MPL in the hematopoietic system is well established, emerging evidence indicates that aberrant c-MPL expression contributes to oncogenic processes in solid tumors, including breast cancer^[^6,7^]^.

HIGHLIGHTS

- Cellular myeloproliferative leukemia (c-MPL) signaling drives breast cancer progression through Janus kinase/signal transducer and activator of transcription, phosphatidylinositol 3-kinase/protein kinase B/mechanistic target of rapamycin (mTOR), and mitogen-activated protein kinase/extracellular signal-regulated kinase (ERK) pathways, enhancing proliferation, survival, stemness, and metastatic potential.
- High c-MPL expression correlates with advanced tumor stage, lymph node metastasis, and poor overall survival.
- c-MPL promotes epithelial–mesenchymal transition, facilitating invasion, dissemination, and therapy resistance.
- Tumor microenvironment modulation via c-MPL, including angiogenesis and platelet interactions, supports metastasis and immune evasion.
- Therapeutic targeting of c-MPL via inhibitors, monoclonal antibodies, or RNA-based strategies offers potential to overcome resistance and limit tumor progression.

In breast cancer, c-MPL is frequently upregulated in aggressive subtypes, such as TNBC and HER2-enriched tumors. Its activation promotes tumor cell proliferation, survival, and the maintenance of cancer stem-like cells, which are associated with therapeutic resistance, recurrence, and metastasis. Beyond tumor-intrinsic effects, c-MPL signaling influences the tumor microenvironment (TME) by stimulating angiogenesis, modulating immune cell infiltration, and facilitating epithelial-to-mesenchymal transition (EMT), collectively fostering tumor progression and dissemination. These observations suggest that c-MPL functions as both a biomarker of aggressive disease and a potential driver of malignancy^[^8–10^]^. Recent preclinical studies highlight the therapeutic potential of targeting c-MPL in breast cancer. Strategies such as monoclonal antibodies that block ligand binding, small-molecule inhibitors that disrupt downstream signaling, and combination therapies with chemotherapy or immunotherapy have demonstrated efficacy in reducing tumor growth, metastasis, and stemness. However, clinical translation remains limited, emphasizing the need for further investigation into c-MPL-mediated signaling pathways, prognostic relevance, and therapeutic vulnerabilities^[^11–14^]^.

This review aims to provide a comprehensive overview of c-MPL signaling in breast cancer, focusing on its molecular mechanisms, interaction with the TME, prognostic implications, and emerging therapeutic strategies. By consolidating current evidence, this review seeks to inform future research and clinical applications, ultimately improving personalized management and outcomes for patients with breast cancer.

### Aim

The primary aim of this review is to provide a comprehensive overview of c-MPL signaling in breast cancer, focusing on its molecular mechanisms, role in tumor progression, and interaction with the TME. Additionally, the review aims to explore the prognostic significance of c-MPL and evaluate emerging therapeutic strategies targeting this receptor. By synthesizing current preclinical and clinical evidence, this review seeks to highlight the potential of c-MPL as both a prognostic biomarker and a therapeutic target, ultimately informing future research and improving clinical management of breast cancer patients.

## Methods

This narrative review was conducted through a comprehensive literature search to synthesize current knowledge on c-MPL signaling in breast cancer. Relevant studies were identified using electronic databases, including PubMed, Scopus, and Web of Science, covering publications up to December 2025. The search strategy included combinations of terms such as “c-MPL,” “thrombopoietin receptor,” “breast cancer,” “signaling pathways,” “tumor microenvironment,” “prognostic biomarker,” and “therapeutic target.”

Inclusion criteria encompassed original research articles, preclinical studies, clinical studies, and relevant review articles that focused on c-MPL expression, signaling mechanisms, prognostic significance, or therapeutic targeting in breast cancer. Articles not published in English or lacking direct relevance to breast cancer were excluded. The selected literature was critically appraised, and key findings were synthesized narratively. Emphasis was placed on molecular mechanisms, clinical correlations, and emerging therapeutic approaches to provide a comprehensive understanding of c-MPL’s role in breast cancer progression and management.

### C-MPL expression in breast cancer

c-MPL, also known as the TPO receptor, is a transmembrane receptor primarily recognized for its role in hematopoietic stem cell maintenance and platelet production. Emerging evidence indicates that c-MPL is aberrantly expressed in several solid tumors, including breast cancer, suggesting a functional role in tumorigenesis beyond its hematopoietic functions. In breast cancer, c-MPL expression has been detected across multiple molecular subtypes, with particularly elevated levels observed in aggressive phenotypes such as triple-negative and HER2-enriched tumors^[^15,16^]^. Immunohistochemical analyses and transcriptomic studies have consistently demonstrated that high c-MPL expression correlates with unfavorable clinicopathological features. These include higher histological grade, increased tumor size, lymphovascular invasion, and positive lymph node status. Moreover, c-MPL expression is often associated with poor overall survival and shorter disease-free survival, positioning it as a potential prognostic biomarker for risk stratification. The receptor’s expression is not uniform across all tumor cells; rather, it is enriched in subpopulations with stem-like characteristics, suggesting a role in maintaining cancer stem cell pools that contribute to therapy resistance and tumor recurrence^[^17,18^]^.

Mechanistically, c-MPL expression in breast cancer allows tumor cells to engage in autocrine and paracrine signaling with TPO or other microenvironmental factors, activating downstream oncogenic pathways such as JAK/STAT, PI3K/AKT, and MAPK/ERK. These pathways promote proliferation, survival, invasion, and metastatic potential. Additionally, c-MPL-expressing tumor cells can modulate the surrounding microenvironment by promoting angiogenesis, immune evasion, and extracellular matrix remodeling, further supporting tumor progression^[^19,20^]^. The clinical relevance of c-MPL expression has also been highlighted in studies examining therapy response. Tumors with elevated c-MPL often display resistance to conventional chemotherapy and targeted agents, underscoring the receptor’s contribution to treatment refractoriness. Consequently, evaluating c-MPL levels in breast cancer tissues could aid in prognostication, guide therapeutic decisions, and identify patients who may benefit from emerging c-MPL-targeted strategies (Table 1)^[^21^]^.

**Table 1:** c-MPL expression in breast cancer.

Sample type	Breast cancer subtype	c-MPL expression	Clinical correlation
Tissue microarray	Triple-negative, HER2+	High in TNBC and HER2-enriched	Associated with higher tumor grade and lymph node metastasis
Immunohistochemistry	ER+, PR+, TNBC	Elevated in aggressive subtypes	Correlated with poor overall survival
RNA-seq analysis	All subtypes	Upregulated in TNBC	Linked to therapy resistance and recurrence
Western blot/IHC	HER2+ and TNBC	High c-MPL expression in 40–50% of cases	Associated with proliferation index (Ki-67) and reduced disease-free survival
qPCR/IHC	Mixed subtypes	Variable, higher in stem-like populations	Predictive of poor prognosis and enhanced metastatic potential

TNBC, triple-negative breast cancer; ER, estrogen receptor; PR, progesterone receptor; HER2, human epidermal growth factor receptor 2; IHC, immunohistochemistry.

### Molecular mechanisms of c-MPL signaling in breast cancer

c-MPL, the receptor for TPO, functions as a critical regulator of multiple intracellular signaling cascades that influence breast cancer progression. Upon ligand binding, c-MPL undergoes dimerization, triggering conformational changes that activate associated intracellular kinases, most notably JAK2. This initial activation sets in motion a network of downstream signaling pathways, including JAK/STAT, PI3K/AKT, and MAPK/ERK, which collectively regulate proliferation, survival, stemness, and metastasis^[^22^]^. The JAK/STAT pathway is one of the primary mediators of c-MPL signaling. Activation of JAK2 leads to phosphorylation of STAT3 and STAT5, which then translocate to the nucleus to induce transcription of genes controlling cell cycle progression, anti-apoptotic factors, and pro-survival proteins. In breast cancer, persistent activation of JAK/STAT by c-MPL supports uncontrolled proliferation, enhances resistance to apoptosis, and contributes to therapy refractoriness. This pathway also plays a key role in maintaining cancer stem-like cells, which are crucial for tumor recurrence and metastasis^[^23^]^.

Parallel activation of the PI3K/AKT pathway further promotes tumor survival and growth. Phosphorylation of AKT enhances metabolic adaptation, inhibits apoptosis, and facilitates resistance to chemotherapeutic agents. Through PI3K/AKT signaling, c-MPL supports cancer stem cell maintenance and contributes to EMT, increasing invasive and metastatic potential^[^24^]^. The MAPK/ERK pathway is another major effector of c-MPL signaling. Engagement of this pathway promotes cell proliferation, differentiation, and motility. In breast cancer, MAPK/ERK activation downstream of c-MPL enhances tumor aggressiveness and facilitates migration and invasion, thereby increasing the likelihood of metastasis to distant organs^[^25^]^. Beyond these canonical pathways, c-MPL signaling also intersects with other cellular processes. For example, it influences angiogenesis by upregulating vascular endothelial growth factor (VEGF) expression, contributing to neovascularization and tumor sustenance. c-MPL can modulate the TME by promoting the recruitment of immunosuppressive cells, such as regulatory T cells and myeloid-derived suppressor cells, thereby facilitating immune evasion. Furthermore, c-MPL-mediated signaling enhances extracellular matrix remodeling, supporting invasion and metastatic dissemination (Table 2)^[^26–28^]^.

**Table 2:** Molecular mechanisms of c-MPL signaling in breast cancer.

Signaling pathway	Mechanism of activation	Functional effects in breast cancer	Clinical implications/therapeutic relevance
JAK/STAT (STAT3/STAT5)	c-MPL dimerization → JAK2 phosphorylation → STAT3/5 activation	Promotes proliferation, survival, anti-apoptotic signaling, and cancer stem cell maintenance	Associated with therapy resistance; targetable with JAK2 inhibitors
PI3K/AKT	c-MPL activation → PI3K → AKT phosphorylation	Enhances cell survival, metabolic adaptation, stemness, EMT, and resistance to chemotherapy	Targetable with PI3K/AKT inhibitors; contributes to metastatic potential
MAPK/ERK	c-MPL activation → RAS → RAF → MEK → ERK phosphorylation	Stimulates proliferation, differentiation, motility, and invasion	Supports tumor growth and metastasis; potential target for MAPK pathway inhibitors
VEGF-mediated angiogenesis	c-MPL signaling upregulates VEGF expression	Promotes neovascularization and nutrient supply to tumors	Enhances tumor progression and metastasis; may synergize with anti-angiogenic therapy
Immune modulation	c-MPL induces recruitment of Tregs and MDSCs; reduces antigen presentation	Creates immunosuppressive TME facilitating immune evasion	Potentially enhances response to immunotherapy when combined with c-MPL targeting
EMT and ECM remodeling	c-MPL signaling induces EMT transcription factors and MMP expression	Increases invasiveness and metastatic dissemination	Supports metastatic progression; therapeutic blockade may reduce metastasis

EMT, epithelial-to-mesenchymal transition; VEGF, vascular endothelial growth factor; TME, tumor microenvironment; Tregs, regulatory T cells; MDSCs, myeloid-derived suppressor cells.

### Interaction with the TME

The TME plays a pivotal role in breast cancer progression, influencing tumor growth, invasion, metastasis, and response to therapy. The TME is composed of a dynamic network of stromal cells, immune cells, extracellular matrix (ECM) components, and soluble factors that interact with cancer cells to regulate tumor behavior. c-MPL signaling has emerged as a critical mediator of these interactions, modulating both tumor-intrinsic and extrinsic processes that facilitate malignancy^[^29,30^]^. One of the key ways c-MPL influences the TME is through angiogenesis. Activation of c-MPL in breast cancer cells enhances the secretion of pro-angiogenic factors, most notably VEGF, which stimulates endothelial cell proliferation and the formation of new blood vessels. This neovascular network not only supplies nutrients and oxygen to the growing tumor but also provides routes for metastatic dissemination^[^31,32^]^. In addition to promoting angiogenesis, c-MPL contributes to immune modulation within the TME. Its signaling promotes the recruitment of immunosuppressive cells, such as regulatory T cells (Tregs) and myeloid-derived suppressor cells, which dampen anti-tumor immune responses. c-MPL activation can also reduce the expression of antigen-presenting molecules and pro-inflammatory cytokines, creating an immunologically “cold” environment that allows tumor cells to evade immune surveillance. These immunosuppressive effects enhance tumor survival and resistance to immunotherapy^[^33,34^]^.

c-MPL also influences stromal interactions and ECM remodeling, processes that facilitate invasion and metastasis. Through the induction of EMT, c-MPL promotes a more motile and invasive phenotype in tumor cells. Concurrently, it upregulates matrix metalloproteinases, enzymes that degrade ECM components, allowing cancer cells to penetrate surrounding tissues and migrate to distant sites^[^35,36^]^. The interplay between c-MPL-expressing tumor cells and the TME underscores the receptor’s multifaceted role in breast cancer progression. By simultaneously driving tumor cell proliferation, stemness, invasion, and metastasis, while shaping a supportive vascular and immunosuppressive microenvironment, c-MPL functions as both an intrinsic oncogenic driver and an extrinsic facilitator of malignancy. Targeting c-MPL may therefore provide dual benefits: direct inhibition of tumor growth and disruption of the supportive TME, enhancing therapeutic efficacy^[^37,38^]^.

### Therapeutic implications of c-MPL in breast cancer

The multifaceted role of c-MPL in breast cancer – promoting tumor proliferation, survival, stemness, metastasis, and modulation of the TME – makes it an attractive candidate for targeted therapy. By disrupting c-MPL signaling, it may be possible to inhibit tumor growth, reduce metastatic potential, overcome therapy resistance, and enhance the efficacy of existing treatments. Several therapeutic strategies are under investigation, ranging from receptor-level inhibition to downstream pathway targeting and combination approaches^[^39,40^]^. Monoclonal antibodies represent a direct approach to targeting c-MPL. These antibodies are designed to bind the receptor, blocking interaction with TPO and preventing activation of downstream signaling pathways. Preclinical studies demonstrate that monoclonal antibodies against c-MPL reduce tumor cell proliferation, induce apoptosis, and impair the self-renewal capacity of cancer stem-like cells. Furthermore, by attenuating c-MPL-mediated angiogenesis and immune suppression, these antibodies may remodel the TME to be less supportive of tumor progression^[^41,42^]^.

Small-molecule inhibitors offer another therapeutic strategy by targeting kinases downstream of c-MPL activation, including JAK2, PI3K, and MAPK. Inhibition of these pathways has been shown to suppress tumor growth, induce apoptosis, and enhance sensitivity to chemotherapeutic agents in breast cancer models. Small-molecule inhibitors may be particularly effective in tumors with high c-MPL expression, where receptor-driven signaling contributes substantially to malignant behavior^[^43,44^]^. Combination therapies hold promise in maximizing therapeutic benefit. Integrating c-MPL inhibitors with conventional chemotherapy, endocrine therapy, or targeted agents may overcome resistance mechanisms mediated by cancer stem cells or pro-survival signaling pathways. Additionally, combining c-MPL-targeted therapies with immunotherapy could enhance anti-tumor immune responses by reversing the immunosuppressive effects of c-MPL signaling within the TME^[^5,45,46^]^. Although clinical trials directly targeting c-MPL in breast cancer are limited, studies in hematologic malignancies provide proof-of-concept that c-MPL-directed therapies can be effective and tolerable. Translating these approaches to solid tumors will require careful consideration of pharmacokinetics, tumor penetration, and potential off-target effects. Importantly, predictive biomarkers such as c-MPL expression levels or activation status may help identify patients most likely to benefit from these interventions^[^47–49^]^.

## Conclusion

c-MPL plays a central role in breast cancer progression by regulating multiple oncogenic pathways, maintaining cancer stem-like cell populations, and shaping a supportive TME. Its aberrant expression is associated with aggressive tumor phenotypes, therapy resistance, and poor clinical outcomes, highlighting its potential as a prognostic biomarker. Beyond prognostication, c-MPL represents a promising therapeutic target, with strategies such as monoclonal antibodies, small-molecule inhibitors, and combination therapies showing potential to suppress tumor growth, reduce metastasis, and enhance treatment efficacy.

By simultaneously influencing tumor-intrinsic signaling and extrinsic microenvironmental factors, c-MPL functions as both a driver of malignancy and a facilitator of tumor progression. Future research should focus on standardizing c-MPL detection, elucidating the complete spectrum of its signaling mechanisms, and advancing clinical trials to validate targeted therapies. Leveraging the prognostic and therapeutic potential of c-MPL may enable more precise patient stratification, optimized treatment strategies, and improved outcomes in breast cancer management.
